# Mechanisms and Assessment of Genotoxicity of Metallic Engineered Nanomaterials in the Human Environment

**DOI:** 10.3390/biomedicines12102401

**Published:** 2024-10-20

**Authors:** Benjamin M. Liu, A. Wallace Hayes

**Affiliations:** 1 Division of Pathology and Laboratory Medicine, Children’s National Hospital, Washington, DC 20010, USA; 2 Department of Pediatrics, George Washington University School of Medicine and Health Sciences, Washington, DC 20010, USA; 3 Department of Pathology, George Washington University School of Medicine and Health Sciences, Washington, DC 20037, USA; 4 Department of Microbiology, Immunology & Tropical Medicine, George Washington University School of Medicine and Health Sciences, Washington, DC 20037, USA; 5 Children’s National Research Institute, Washington, DC 20012, USA; 6 The District of Columbia Center for AIDS Research, Washington, DC 20052, USA; 7 Center for Environmental/Occupational Risk Analysis & Management, University of South Florida College of Public Health, Tampa, FL 33612, USA; 8 Institute for Integrated Toxicology, Michigan State University, East Lansing, MI 48824, USA

**Keywords:** engineered nanomaterials, metallic, genotoxicity, mechanisms, assessment, guidance, DNA damage

## Abstract

Engineered nanomaterials (ENMs) have a broad array of applications in agriculture, engineering, manufacturing, and medicine. Decades of toxicology research have demonstrated that ENMs can cause genotoxic effects on bacteria, mammalian cells, and animals. Some metallic ENMs (MENMs), e.g., metal or metal oxide nanoparticles TiO_2_ and CuO, induce genotoxicity via direct DNA damage and/or reactive oxygen species-mediated indirect DNA damage. There are various physical features of MENMs that may play an important role in promoting their genotoxicity, for example, size and chemical composition. For a valid genotoxicity assessment of MENMs, general considerations should be given to various factors, including, but not limited to, NM characterization, sample preparation, dosing selection, NM cellular uptake, and metabolic activation. The recommended in vitro genotoxicity assays of MENMs include *hprt* gene mutation assay, chromosomal aberration assay, and micronucleus assay. However, there are still knowledge gaps in understanding the mechanisms underlying the genotoxicity of MENMs. There are also a variety of challenges in the utilization and interpretation of the genotoxicity assessment assays of MENMs. In this review article, we provide mechanistic insights into the genotoxicity of MENMs in the human environment. We review advances in applying new endpoints, biomarkers, and methods to the genotoxicity assessments of MENMs. The guidance of the United States, the United Kingdom, and the European Union on the genotoxicity assessments of MENMs is also discussed.

## 1. Introduction

Based on the 2022 European Commission (E.C.) Recommendation [1], a nanomaterial (NM) is defined as “a natural, incidental or manufactured material consisting of solid particles that are present, either on their own or as identifiable constituent particles in aggregates or agglomerates, and where 50% or more of these particles in the number-based size distribution fulfill at least one of the following conditions: (a) one or more external dimensions of the particle are in the size range 1 nm to 100 nm; (b) the particle has an elongated shape, such as a rod, fiber or tube, where two external dimensions are smaller than 1 nm, and the other dimension is larger than 100 nm; (c) the particle has a plate-like shape, where one external dimension is smaller than 1 nm and the other dimensions are larger than 100 nm”. In contrast to those existing and occurring naturally, engineered nanomaterials (ENMs) are the NMs engineered to acquire special optical, magnetic, electrical, and other properties [2,3,4].

ENMs have a broad array of applications in agriculture, engineering, manufacturing, and medicine. Nanotechnologies have been widely used in medical imaging, e.g., magnetic resonance imaging, computerized tomography scans, positron emission tomography scans, and point-of-care diagnostic tests aiding in diagnosing infectious diseases, cancers, and other illnesses [4]. Compared with traditional research and diagnostic tools (e.g., cultures, antigen testing, serologic testing, and PCRs) [5,6,7,8,9,10,11,12,13], biosensors with nanotechnology and nanopore sequencing allow for the rapid, sensitive, and accurate detection of biomarkers, DNA, or RNA molecules [4,14,15,16,17,18,19,20,21,22,23]. NM-synergized photodynamic therapy and photothermal therapy have developed into efficient and non-invasive treatment modalities for fighting different types of cancer [24]. NMs can be manipulated to generate specialized drug delivery systems that are important for the elimination of metastatic tumor cells when being utilized in a combination of phototherapy and immunotherapy [24].

However, there has been increasing concern about environmental contamination, biosafety, and the potential adverse effects of ENMs on human health and the environment [25,26,27]. The main sources of ENMs in the natural and human environment can be found in air (atmosphere), water (hydrosphere), and soil (lithosphere), including but not limited to industrial emissions, ENM waste disposal, and NMs released from consumer products [28]. There are numerous paths of exposure to ENMs, including, but not limited to, occupational exposure, oral exposure, and skin exposure (e.g., contact with textiles, cosmetics, food packaging, etc.) [29,30]. For example, carbon-based NMs consisting of carbon nanotubes are widely used in microelectronics, nanomedicine, and hydrogen fuel that can lead to exposures comparable to asbestos [27]. Graphene oxide, an oxidation derivative of graphene, may also lead to human exposure and biosafety concerns [27].

Decades of toxicology research have demonstrated that ENMs can cause genotoxic effects on bacteria, mammalian cells, and animals, which may differ from materials containing the same elements but are due to their special physical and chemical features [27,31]. For example, metal oxide nanoparticles (NPs) can exert clastogenic effects on the DNA of living organisms by inducing DNA strand or chromosomal breaks and micronucleus formation [32]. Another appealing example is the toxicity of artificial NMs to animals. Nano La_2_O_3_ leading to oxidative stress and the subsequent failure of the antioxidant system was demonstrated to be highly detrimental to *Eisenia fetida* [33]. Nanoparticulate TiO_2_ and ZnO, important components of food packaging and containers and toothpaste, can induce cytotoxicity and genotoxic responses and have led to public concerns about oral exposure to metallic ENMs (MENMs) in our daily lives [26,34,35]. MENMs are a class of ENMs made of metal or metal oxide NPs that are employed in a variety of consumer products to improve their electrical, optical, and magnetic properties [32,34]. However, there are still knowledge gaps in understanding the mechanisms underlying the genotoxicity of MENMs and utilizing novel genotoxicity biomarkers. There are also a variety of challenges in the utilization and interpretation of MENM genotoxicity assessment assays.

In this review article, we provide mechanistic insights into the genotoxicity of MENMs in the human environment. We review advances in applying new endpoints, biomarkers, and methods to the genotoxicity assessments of MENMs. The guidance of the United States, the United Kingdom, and the European Union on the genotoxicity assessments of nanomaterials is also discussed. Therefore, the relevance and purpose of this review article is to provide mechanistic insights into the genotoxicity of MENMs; discuss endpoints, biomarkers, and assessment methods for MENM-associated genotoxicity; review guidance on the genotoxicity assessments of NMs; and provide perspectives on future research directions.

## 2. Physical and Chemical Properties of NPs 

ENMs have multiple physical and chemical features that can promote genotoxicity, including source, size, shape, surface properties, surface charges, aggregation/agglomeration state, quality, chemical structures, and nanoparticle purity. Among them, chemical structure, size, and chemical composition are the most important properties associated with their impact on human health [27,29].

First, chemical structure is one of the important chemical properties of NPs, based on which NPs can be classified as organic and inorganic [27,29]. Inorganic NPs include metals, metal oxides, etc., whereas organic NPs include carbon NPs (fullerenes, graphenes, etc.), nanoplastics (including rubber nanoparticles), etc. [27,29]. We focus on MENMs in this review.

Second, the size of NMs is related to their ability to penetrate different cells or biological membranes and cause mechanical interference with cellular components. NMs smaller than 100 nm can easily penetrate cells, and those with a size of 40 nm can cross into the nuclei of cells, while those below 35 nm pass through the blood–brain barrier (BBB) [27]. Smaller NPs are more toxic than the larger ones based on the following reasons: First, it is much easier for smaller NPs to cross cellular membranes. Smaller NPs can more easily cross cellular membranes and reach the nucleus. Second, with a larger surface area and higher surface area to volume ratio, smaller NPs are more chemically reactive. Smaller NPs have a higher likelihood to interact with cellular organelles and DNA, thereby more easily causing DNA damage.

Moreover, the chemical composition of the MENMs is an important parameter that should be considered when testing for the potential genotoxicity of an MENM. For example, Sahu and his colleagues demonstrated that Ag NMs can lead to genotoxicity and cytotoxicity in human liver cells [36]. TiO_2_ can induce cytotoxicity after entering the cytoplasm, significantly increasing oxidative stress and DNA oxidative damage in human normal bronchial epithelium BEAS-2B cells [35]. Vieira et al. [37] determined the intracellular reactive oxygen species (ROS) induction and the genotoxicity of three different TiO_2_ NMs (NM-102, NM-103, and NM-105) by using human colorectal adenocarcinoma cells Caco-2 and human colon intestinal cells HT29-MTX-E12. They demonstrated that the DNA-damaging effect was dependent on the NM type, possibly because of their smaller hydrodynamic size in the cell medium [37].

In addition, the increased reactivity of MENMs may also occur due to increased surface area, surface properties, increased absorption, the ability to reach deep airways, dermal penetration, systemic distribution, penetration of the BBB or placental barriers, and biological persistence [29,38,39]. Guo et al. recently reported that NPs purified from groundwater with multiple enzymatic activities can efficiently cross the BBB [39]. It is believed that AuNPs can induce oxidative stress in vitro and in vivo in brain cells [38]. Though NMs are generally considered insoluble in aqueous media [31], the concentration and solubility of the dissolved species play an important role in the ZnO-induced genotoxic response and CuO-mediated genotoxicity, suggesting that the particles and their dissolved fractions may exert a differential impact on the genotoxicity induced by metal oxide nanomaterials (MONMs) [34]. CuO NPs, ZnO NPs and microparticles, and ZnCl_2_ exposures have been demonstrated to induce dose- and time-dependent increases in DNA damage [34].

## 3. Mechanistic Insights into the Genotoxicity of MENMs

Increasing evidence supports that MENMs exert genotoxic effects, e.g., oxidative DNA damage and point mutations, on plants, mammals, and bacteria, which even lead to altered gene expression [29,37]. The mechanisms of action of MENMs, even at low doses, have been progressively characterized by employing omics approaches and systems toxicology applied in nanotoxicology studies [38]. Gold NPs (AuNPs) with variable ranges of particle size and shape have been demonstrated to trigger a broad array of biological responses, leading to omics changes (transcriptomics, proteomics, metabolomics, epigenomics, and epitranscriptomics) in human and mouse cells [38,40].

**Figure 1:**
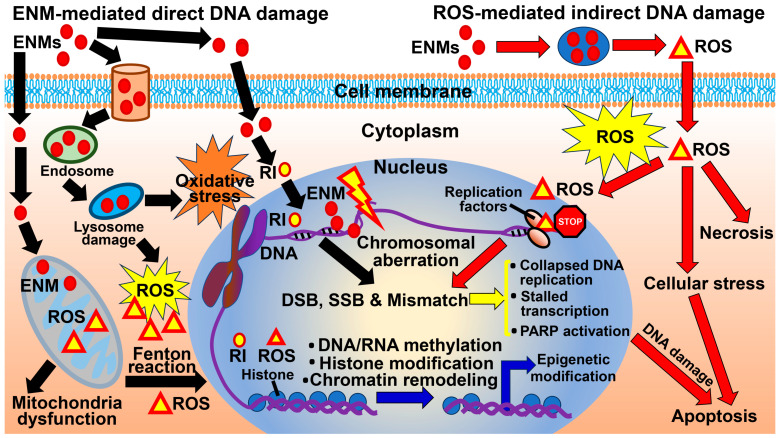
Mechanisms underlying the genotoxicity of ENMs, e.g., MENMs. ENMs induce genotoxicity via two mechanisms, i.e., direct DNA damage (**left**) and reactive oxygen species (ROS)-mediated indirect DNA damage (**right**). (**Left**, black arrows) small MENMs, e.g., those with a size of 40 nm, can cross into the nuclei of cells and cause direct DNA damage, thereby leading to genotoxic effects. Some MENMs can be endocytosed, cause lysosome damage, and lead to oxidative stress, thereby generating reactive ions (RI). Some MENMs crossing the cell membrane can cause mitochondria dysfunction and trigger a Fenton reaction, thereby generating ROS. Both MENMs and RI entering the nucleus can cause chromosomal aberration. (**Right**, red arrows) MENMs that enter and accumulate in the cytoplasm can trigger biochemical reactions to generate ROS, which is considered the underlying mechanism involved in the genotoxicity of metal oxide NPs. (Yellow arrows) both direct and indirect DNA damage pathways can lead to double-strand DNA breaks, single-strand breaks, and mismatches, thereby altering DNA replication, stalling transcription, and PARP activation. ROS can cause necrosis, cellular stress, and apoptosis. DNA damage can also cause apoptosis. (Blue arrows) MENMs and ROS may also induce epigenetic changes via DNA or RNA methylation, histone modification, and chromatin remodeling. PARP, poly(ADP-ribose) polymerase; DSB, double-strand DNA break; SSB, single-strand breaks; RI, reactive ion; ROS, reactive oxygen species.

MENMs can induce genotoxicity via at least two mechanisms, i.e., direct DNA damage (Figure 1, left) and ROS-mediated indirect DNA damage (Figure 1, right), in different models, cell types, and organisms, including mammalian cells, human cell lines, and aquatic vertebrate models [29,36,39,41]. On the one hand, small MENMs, e.g., those with a size of 40 nm, can cross into the nuclei of cells and cause direct DNA damage, thereby leading to genotoxic effects (Figure 1, left) [29]. Some MENMs can be endocytosed, cause lysosome damage, and lead to oxidative stress, thereby generating reactive ions (RIs), which are chemically reactive ions that can cause chemical or biological reactions, e.g., hydrogen ions and metal ions (Figure 1, left). Some MENMs crossing the cell membrane can cause mitochondria dysfunction and trigger a Fenton reaction, thereby generating ROS (Figure 1, left) [42]. Both MENMs and RIs entering the nucleus can cause chromosomal aberration (Figure 1) [42]. On the other hand, MENMs that enter and accumulate in the cytoplasm can trigger biochemical reactions to generate ROS, which is considered the underlying mechanism involved in the genotoxicity of metal oxide NPs [29] (Figure 1, right). Both the direct and indirect DNA damage pathways can lead to double-strand DNA breaks, single-strand breaks, and mismatches, thereby altering DNA replication, stalling transcription, and poly(ADP-ribose) polymerase activation (Figure 1, bottom) [42]. ROS can cause necrosis, cellular stress, and apoptosis, whereas DNA damage can also cause apoptosis (Figure 1) [29]. MENMs and ROS may also induce epigenetic changes via DNA or RNA methylation, histone modification, and chromatin remodeling (Figure 1, bottom) [29]. The omics changes due to the AuNP exposure were caused partially by the ROS activation and oxidative stress generated [38].

## 4. Endpoints and Biomarkers for Genotoxicity

With the development of cell biology, molecular biology, microbiology, and immunology [43,44,45,46,47], new molecular targets, endpoints (e.g., a targeted outcome or event that can be measured objectively), and biomarkers (e.g., molecular biomarkers, e.g., hallmark point mutations) have been applied to research and medicine [48,49,50,51,52,53,54,55,56,57,58,59,60,61,62,63,64,65,66,67,68]. Different biomarkers for genotoxicity, e.g., DNA damage, gene mutations, and chromosomal damage, have been used in hazard assessment in nanotoxicology [69]. For example, 8-Hydroxy-2′-deoxyguanosine (8-OHdG) is a critical marker of oxidative DNA damage and a biomarker for oxidative stress [42,70]. New endpoints and biomarkers have recently been applied to the investigation of various molecular pathways, autophagy, apoptosis, subcellular localization, and epigenetic alterations that are considered implicated in MENM-induced genotoxic effects [71,72]. Table 1 summarizes mechanisms of action and key biomarkers for ENM-associated genotoxicity. For example, DNA oxidative damage is regarded as a more sensitive genetic endpoint to examine the TiO_2_-induced genotoxicity than the standard comet assay [35]. There was a dose-dependent increase in the intracellular levels of ROS and a significant decrease in the ratios between glutathione and oxidized glutathione [35]. Oxidative stress and genotoxicity induced by TiO_2_ NPs were confirmed using the comet assay in conjunction with the enzyme formamidopyrimidine glycosylase [35]. Aquatic vertebrate models, such as the Nile tilapia (*Oreochromis niloticus*), have been used to assess nano-TiO_2_-induced genotoxicity and histological lesions [41]. The ongoing research to develop new assays to assess the novel biomarkers associated with the genotoxicity of MENMs is of paramount importance, ensuring the safe development and application of nanotechnology and the protection of human health and the environment.

## 5. Methods for Genotoxicity Assessment of MENMs

NMs constitute challenges in evaluating their safety as they have a small size, and relatively large surface area, as well as unknown distribution and prevalence in biological systems and the environment [27,31,37,73]. Both the U.S. Food and Drug Administration (FDA) and the Organization for Economic Co-operation and Development emphasize that it may be necessary to evaluate whether traditional standard genotoxicity methods apply to MENMs [27,31,35,37,73,74]. Table 2 shows the main advantages and limitations of methods for the genotoxicity assessment of MENMs. Unlike the interactions between most chemicals and environmental mutagens and host cells, the interactions of NMs with organisms depend upon numerous contributing factors, such as size effect and high surface activity. These unique features of MENMs make some current routinely used genotoxicity standardized methods ineffective and unreliable for NMs, which may lead to conflicting research conclusions [31,35,74]. For example, the Ames test using the tester strain *Salmonella typhimurium* is not recommended for testing MENMs that are bactericidal or impermeable to the bacterial cell wall [31].

**Figure 2:**
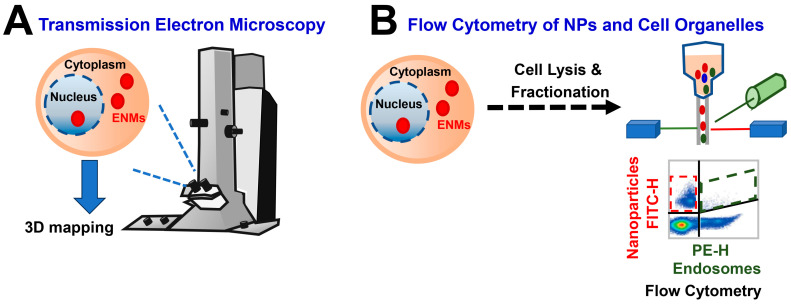
Commonly used test methods for the cellular uptake of ENMs. (**A**) Transmission electron microscopy to visualize the cellular distribution of MENMs inside cells and 3D mapping to ascertain if the particles simply are on top of the cell or internalized. (**B**) Flow cytometry to quantify NP uptake in cell organelles (e.g., endosomes and lysosomes).

In a recent “Common Consideration” paper [31], Elespuru et al. emphasized that general considerations should be given to the characterization of the NMs to be tested, sample preparation, dosing selection, assessment of exposure, uptake of NMs into target cells, positive and negative controls, metabolic activation of genotoxins/carcinogens, and data analysis when one is performing an NM genotoxicity assessment. Among these parameters, the characterization of MENM cellular uptake and distribution is a significant consideration as this valuable information indicates cell exposure and is critical for verifying the validity of NM test results [31,75]. Figure 2 lists two commonly used test methods for the cellular uptake of MENMs, i.e., transmission electron microscopy (TEM) (Figure 2A) and flow cytometry (Figure 2B). By performing TEM, Chen et al. recently demonstrated that TiO_2_ NPs accumulate in the cytoplasm but do not enter the nuclei of human bronchial epithelial cells (BEAS-2B) [35]. Moreover, fluorescence microscopy methods can be used to assess the penetration of NPs [76]. Several NMs are characterized by intrinsic fluorescence, or can be labeled with a fluorescent dye, allowing the visualization of the distribution and penetration depth of the NPs of interest through fluorescence-related microscopy, or laser scanning confocal microscopy [76]. In addition, Garcia Romeu et al. developed and validated a time- and space (intracellular)-resolved flow cytometry of cell organelles (e.g., endosomes and lysosomes) to quantify NP uptake and intracellular trafficking by cells, which demonstrated that this method is a useful addition to a suite of flow cytometry assays for the high-throughput analysis of NP uptake and intracellular trafficking by cells [77] (Figure 2B).

**Figure 3:**
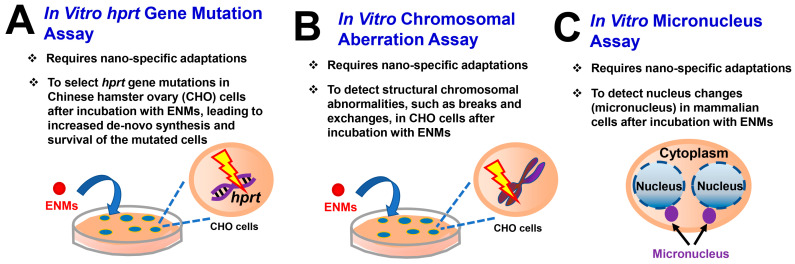
Commonly used in vitro genotoxicity assays of NPs and ENMs. Nanomaterial Genotoxicity Testing Roadmap by Elespuru et al. [31,73] recommended three in vitro genotoxicity assays of NPs and MENMs, i.e., the *hprt* gene mutation assay, chromosomal aberration assay, and micronucleus assay. The principle of these three recommended assays is illustrated. All these assays require nano-specific adaptations to facilitate testing with NPs. (**A**) In vitro *hprt* gene mutation assay detects the ability of MENMs to induce mutations in the hprt gene in Chinese hamster ovary (CHO) cells, which leads to increased de novo synthesis and the survival of the mutated cells. (**B**) In contrast, in vitro chromosomal aberration assay detects structural chromosomal abnormalities, such as breaks and exchanges, in CHO cells after incubation with MENMs. (**C**) Similarly, in vitro micronucleus assay detects nucleus changes in mammalian cells after incubation with MENMs.

A variety of genotoxicity assessment tests have been employed to evaluate the potential genotoxicity of NMs, for example, single cell gel electrophoresis (SCGE) assay (also known as the comet assay), cytokinesis-block micronucleus cytome (CBMN cyt) assay, 8-hydroxydeoxyguanosine DNA adducts detection, hypoxanthine–guanine phosphoribosyltransferase (*hprt*) gene forward mutation assay, and γH2AX staining [31,73,78]. Adding in vivo assessments would be warranted if there is specific organ exposure or the sequestration of NMs. With the introduction of the 3R (Replacement, Reduction, and Refinement) principle, the reduction in animal usage calls for the development of novel in vitro assays for the assessment of MENM-induced genotoxic effects [79]. Figure 3 illustrates the three recommended in vitro genotoxicity assays of NPs and MENMs, i.e., in vitro *hprt* gene mutation assay (Figure 3A), in vitro chromosomal aberration assay (Figure 3B), and in vitro micronucleus assay (Figure 3C), which were included in NM Genotoxicity Testing Roadmap by Elespuru et al. [31,73]. Of note, these assays detect different aspects of the genotoxicity of MENMs [41]. Specifically, in vitro *hprt* gene mutation assay detects the ability of MENMs to induce mutations in *hprt* gene in Chinese hamster ovary (CHO) cells after incubation with MENMs, which leads to increased de novo synthesis and the survival of the mutated cells (Figure 3A) [73]. In contrast, in vitro chromosomal aberration assay detects structural chromosomal abnormalities, such as breaks and exchanges, in CHO cells after incubation with MENMs (Figure 3B), whereas in vitro micronucleus assay detects nucleus changes in mammalian cells (Figure 3C) [73]. Human lymphocytes were also used [73]. Vieira et al. recently performed a micronucleus assay to examine the effects of TiO_2_ NMs on chromosomal integrity in human colon intestinal cells HT29-MTX-E12, in which significantly increased micronucleus frequency was observed after exposure to a low (1.4 μg/mL) and high (14 μg/mL) concentration of digested TiO_2_ NMs [37].

**Figure 4:**
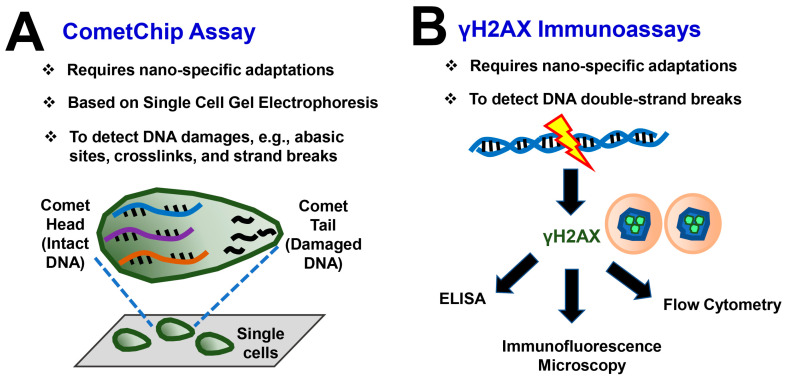
Principles of two high-throughput genotoxicity assays of NPs and ENMs. All these assays require nano-specific adaptations to facilitate testing with NPs. (**A**) CometChip is a high-throughput 96-well platform to aid in the detection of DNA damage (e.g., abasic sites, crosslinks, and strand breaks) in microarrayed single cells based upon the well-established single-cell gel electrophoresis assay (also known as the comet assay). After migration through a matrix in an electrical field, the relative size of the comet tail (containing damaged DNA) is an index of DNA damage, which facilitates estimating the level of DNA damage. (**B**) The formation of γH2AX after the phosphorylation of histone variant H2AX represents a primary response to DNA damage. γH2AX serves as a useful biomarker with a high sensitivity and robustness for the detection of DNA double-strand breaks, and there are various modalities of immunoassays suitable for the γH2AX detection, including, but not limited to, Western blot, ELISA, flow cytometry, and immunofluorescence microscopy.

Notably, high-throughput in vitro assays can provide an expedited analysis of the induced toxicity of MONMs while minimizing the overall use of animals [34]. As shown in Figure 4A, the CometChip is a high-throughput 96-well platform to aid in the detection of DNA damage (e.g., abasic sites, crosslinks, and strand breaks) in microarrayed single cells based upon the well-established SCGE. After migration through a matrix in an electrical field, the relative size of the comet tail (containing damaged DNA) is an index of DNA damage, which facilitates estimating the level of DNA damage. Boyadzhiev et al. [34] employed the high-throughput CometChip assay to assess the in vitro genotoxic effects of CuO, ZnO, and TiO_2_ MONMs and microparticles, as well as five coated/surface-modified TiO_2_ NPs and ZnCl_2_ and CuCl_2_ after 2-4 h of exposure. As a result, dose- and time-dependent DNA damage was detected after exposure to the CuO NPs, ZnO NPs and MPs, and ZnCl_2_ at different time points [34]. In addition, the formation of γH2AX after the phosphorylation of histone variant H2AX represents a primary response to DNA damage [80] (Figure 4B). γH2AX serves as a useful biomarker with a high sensitivity and robustness for the detection of DNA double-strand breaks, and there are various modalities of immunoassays suitable for the γH2AX detection, including, but not limited to, Western blot, ELISA, flow cytometry, and immunofluorescence microscopy (Figure 4B) [80,81].

With their advent in recent years, novel technologies such as three-dimensional (3D) models of skin, lung, and liver tissues have been used in assays using complex approaches to examining the biomarkers of genotoxic effects [82]. For example, a series of 3D tissue models coupled with a variety of genotoxicity assessment tools have been established, including skin micronucleus test (RSMN) and RS comet assay, comet assays using two commercially available human reconstructed 3D airway models, and 3D HepG2 liver spheroids with CBMN assay [82]. The RSMN test using 3D skin models is considered an acceptable alternative to the in vivo test, with an overall assay accuracy of 84%, sensitivity of 80%, and specificity of 87% when compared to in vivo genotoxicity outcomes. While showing promise, liver and airway model-based genotoxicity assays are at an early stage of development [82].

## 6. Guidance of the United States, the United Kingdom, and the European Union on the Genotoxicity Assessments of NMs

In the United States, multiple federal agencies are tasked with overseeing and regulating ENMs and the applications of nanotechnology. In June 2014, the U.S. FDA published Guidance for Industry Safety of Nanomaterials in Cosmetic Products, a guidance document regarding FDA-regulated products involving the application of nanotechnology [83]. In this guidance document, an overarching framework was provided to describe the FDA’s approach to regulating products involving the application of nanotechnology. Based on the current understanding of the scientific and technical characteristics of NMs, the FDA believes that any unique properties and behaviors that may be impacted by the application of nanotechnology should be considered when evaluating the safety, efficacy, possible impact on public health, or regulatory status of nanotechnology products [83]. Two Points to Consider were identified that should be used when evaluating whether the application of nanotechnology is involved in the FDA-regulated products [83]. Both particle dimensions and dimension-dependent properties or phenomena are addressed in these points. The first point to consider is related to a material’s or end product’s particle dimensions [83]. Specifically, the FDA will ask whether there is at least one external dimension or an internal or surface structure in an engineered material or end product in the nanoscale range (approximately 1 nm to 100 nm). The second point to consider is related to dimension-dependent properties or phenomena of an engineered material or end product. The FDA will ask whether they exhibit dimension-dependent physical or chemical properties or biological effects [83]. These considerations apply not only to new products but also to FDA-regulated products or any of their constituent parts with changes to manufacturing processes that alter their dimensions, properties, or effects [83]. The FDA recommended that the safety assessment for cosmetic products using nanomaterials should address impurities, the physicochemical characteristics, the potential routes of exposure to the NMs, the potential for the aggregation and agglomeration of NPs in the final product, dosimetry for in vitro and in vivo toxicology studies, and in vitro and in vivo toxicological effects [84].

In early 2012, the UK’s Committee on Mutagenicity of Chemicals in Food, Consumer Products, and the Environment (COM) published its review of the genotoxicity assessment of NMs and experimental considerations [85]. The COM concluded that standard protocols could not be used directly in testing NMs as those applied to the genotoxicity testing of chemicals in general [85]. However, each test would need to have a characterization of the physicochemical features of the NMs and a demonstration of their uptake into cells. Gene mutations in mammalian cells (*hprt* gene mutation assay) and the in vitro micronucleus test were considered the most appropriate in vitro package. The COM concluded that bacterial gene mutation tests may not be suitable for NMs because of the penetration issues of these particles into bacterial cell walls and the inability of bacterial cells to phagocytose particles [85]. The COM agreed that oxidative DNA damage has been found in the genotoxicity testing of many NMs and that aneuploidy may be induced by carbon nanotubes after interaction with the mitotic spindle apparatus [85].

The genotoxicity assessment of NMs was addressed in 2018 by a working group of the International Life Sciences Institute (ILSI) Health and Environmental Sciences Institute (HESI, Washington, DC, USA) Genetic Toxicology Technical Committee (GTTC) [73]. The assays reviewed by the working group included the bacterial reverse mutation (Ames) assay; in vitro mammalian assays for mutations, chromosomal damage, micronucleus induction, or DNA strand breaks; and in vivo assays for genetic damage in various target tissues (micronucleus, comet, and transgenic mutation assays) [73]. The working group recommended that in vitro mammalian cell mutagenicity and clastogenicity assessments be included in a revised test battery for NM genotoxicity [73]. Adding in vivo assessments would be warranted if there is specific organ exposure or the sequestration of NMs. The revised genotoxicity assessment test battery for NMs does not recommend bacterial assays as the bacteria-based assays face some problems, for example, suboptimal particle uptake [73]. Other recommendations include the characterization of NMs in the test medium and the assessment of the uptake of NMs into target cells and their cellular distribution [73].

In Europe, the EU Cosmetic Regulation provides a definition of NMs and specifically covers the regulation of the use of NMs in cosmetics, which requires the premarket notification, safety evaluation, and labeling of NMs intended for use in cosmetic products [86]. On 10 June 2022, a recommendation on the definition of NMs was issued by the European Commission for legislative purposes. This document (Commission Recommendation of 10.06.2022 on the definition of nanomaterial) provided an update to the previous definition that was made in the Recommendation 2011/696/EU [1].

**Figure 5:**
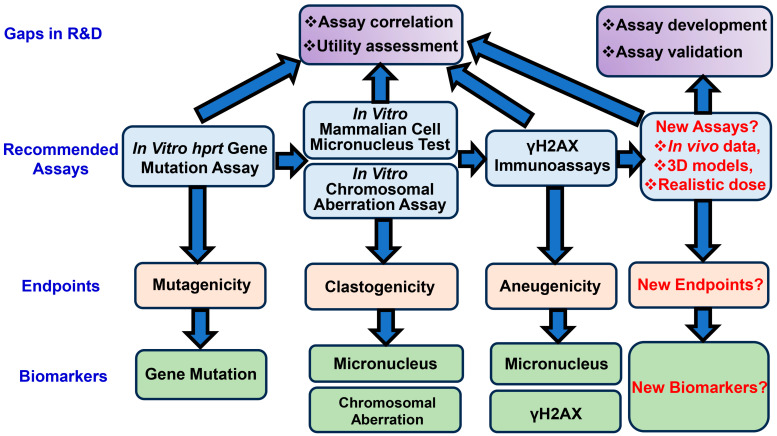
Current algorithm and future perspectives of assessment assays for MENM-associated genotoxicity. (**Second row**) Recommended assessment assays for MENM-associated genotoxicity include in vitro mutagenicity assay (e.g., in vitro *hprt* gene mutation assay), in vitro mammalian cell micronucleus test, in vitro chromosomal aberration assay, and γH2AX immunoassays. These assays are used to detect the corresponding endpoints (**third row**) and biomarkers (**bottom**). (**Right**) new assays, endpoints, and biomarkers are needed for a better assessment of MENM-associated genotoxicity. (**Top row**) there are gaps in R&D for the current and new assays.

The Scientific Committee on Consumer Safety (SCCS) provides the European Commission with scientific advice on the safety of non-food consumer products and up-to-date guidance on the safety assessment of NMs in cosmetics. In October 2019, the SCCS adopted and issued the Guidance on the Safety Assessment of NMs in Cosmetics, which covered safety considerations, physicochemical characterization, exposure assessment, hazard identification, dose–response characterization, and risk assessment [86]. The Cosmetic Regulation (EC) No 1223/2009 provides guidance on the risk assessment of NMs used in cosmetic products. It requires the safety assessment of safety data with special considerations of the properties of that specific NM. This regulation applies to any new or already approved ingredient when meeting the criteria for defining an NM [86]. It is suggested by SCCS that in vitro genotoxicity tests be conducted for NMs, including mammalian cell chromosome aberration/clastogenicity tests (e.g., micronucleus test), an in vitro mammalian cell gene mutation test (e.g., *hprt* test), or other indicator tests (e.g., the comet assay). Figure 5 summarizes the current algorithm of assessment assays for MENM-associated genotoxicity, including the above-mentioned assays.

## 7. Conclusions

Despite having broad applications in agriculture, engineering, manufacturing, and medicine, MENMs have been regarded as an emerging class of contaminants due to their environmental bioaccumulation, biosafety concerns, and potential adverse effects on human health and the environment [87,88,89,90,91,92,93]. MENMs have multiple physical features that can promote their genotoxicity, including source, size, shape, surface properties, surface charges, aggregation/agglomeration state, quality, and nanoparticle purity. MENMs have been demonstrated to induce genotoxicity after entering the cytoplasm and nuclei of cells, leading to significantly increased oxidative stress, DNA oxidative damage, point mutations, and altered gene expression. For a valid genotoxicity assessment of MENMs, general considerations should be given to various factors, including, but not limited to, NM characterization, sample preparation, dosing selection, NM cellular uptake, and metabolic activation. The recommended in vitro genotoxicity assays of NPs and MENMs include the *hprt* gene mutation assay, chromosomal aberration assay, and micronucleus assay. Adding in vivo assessments would be warranted if there is specific organ exposure or the sequestration of NMs. New endpoints, biomarkers, and methods have recently been applied to investigate various molecular pathways, autophagy, apoptosis, subcellular localization, and epigenetic alterations implicated in MENM-induced genotoxic effects.

The U.S. FDA identified particle dimensions and dimension-dependent properties or phenomena as two Points to Consider that should be used when evaluating whether the application of nanotechnology is involved in FDA-regulated products. The UK’s COM publishes its review of the genotoxicity assessment of NMs and experimental considerations. In Europe, the EU Cosmetic Regulation provides a definition of an NM and specifically covers the regulation of the use of NMs in cosmetics, which requires the premarket notification, safety evaluation, and labeling of NMs intended for use in cosmetic products. The SCCS provides the European Commission with up-to-date guidance on the safety assessment of NMs in cosmetics. Further research is needed to develop new assays to assess novel biomarkers associated with the genotoxicity of ENMs, ensuring the safe development and application of nanotechnology and the protection of human health and the environment.

## 8. Perspectives

It is of paramount importance to ensure the safe application of MENMs and protect human health and the environment. There is an unmet need to develop new assays for the assessment of novel biomarkers and endpoints associated with the genotoxicity of MENMs (Figure 5, right). More research is needed to accumulate in vivo genotoxicity data on the realistic doses of NEM to facilitate assessments and the management of environmental and human health risks (Figure 5). Further investigation is warranted for the optimization and validation of 3D liver and airway model-based assays to address the lack of coverage of the three main endpoints of genotoxicity (mutagenicity, clastogenicity, and aneugenicity) and on metabolic competence (Figure 5) [94]. It would be important to correlate data from different models and cell types to enhance the commutability of the various testing systems, for example, a comparison between the testing data of aquatic vertebrate models and those for mammalian cells (Figure 5, top). The assessment of the utility of the current and new assays is also warranted (Figure 5, top).

## Figures and Tables

**Table 1:** Mechanisms of action and key biomarkers for ENM-associated genotoxicity.

Genotoxicity Assays	Mechanisms of Action/Endpoints	Biomarkers	Cell Models	NPs to Be Tested
In vitro *hprt* gene mutation assay	Mutagenicity	Gene mutations	Chinese hamster ovary (CHO)	NPs that cause point mutations
In vitro mammalian cell micronucleus test	Clastogenicity	Micronucleus	Mammalian cells	NPs that cause micronucleus
In vitro chromosomal aberration assay	Clastogenicity	Chromosomal aberrations	CHO	NPs that cause chromosomal aberrations

**Table 2:** Main advantages and limitations of methods for genotoxicity assessment of MENMs.

Genotoxicity Assays	Advantages	Limitations
In vitro *hprt* gene mutation assay	Suitable to test MENMs that cause point mutations Simple Robust and cost-effective	Lack of validation data and inconsistent protocols May require specialized reagents Some cell lines used in this assay lack metabolic capacity Fails to cover all mutational mechanisms
In vitro mammalian cell micronucleus test	Simple endpoint that is easy to detect and ensures accuracy Can detect spindle poisons Micronucleated cells are usually stable Any dividing cell population can be used regardless of its karyotype Responses for a longer duration can be detected	Fails to classify chromosomal aberrations Pseudo-micronucleus may be detected in some circumstances Requires careful cell preparation, treatment, fixation, spreading, staining, and/or scoring Can be affected by NPs with fluorescence and adsorption capacity
In vitro chromosomal aberration assay	Suitable to test MENMs that cause chromosomal aberration Simple Robust and cost-effective	Low sensitivity when assessing low-dose exposure compared to other methods False positivity due to aberrations that can occur due to toxicity but are not relevant to human risk Requires expertise and experience

## Data Availability

Not applicable.
